# Oral Health Education, Knowledge, and Practice Patterns of Nurses Caring for Cancer Patients: A Scoping Review

**DOI:** 10.1177/15271544251322765

**Published:** 2025-03-25

**Authors:** Rachael M. Dvorski, Megan V. Hynes, Elise D. Paisley, Shauna M. Hachey

**Affiliations:** 1 School of Dental Hygiene, Faculty of Dentistry, 3688 Dalhousie University, Halifax, Nova Scotia, Canada; 2 210547 Healthy Populations Institute (HPI), 3688 Dalhousie University, Halifax, Nova Scotia, Canada

**Keywords:** cancer, oncology, carcinoma, malignancy, tumor, neoplasm(s), neoplastic, lymphoma, nursing, oncology nursing, nurses, oral health, mouth diseases, oral manifestations, mouth, teeth, saliva, palate, mucosa, gingival and gums

## Abstract

**Background:** Global cancer diagnoses are increasing, and treatment often results in oral health concerns. To improve patient outcomes and quality of life, nurses play a critical role in managing the oral sequelae of treatment. **Aims:** This scoping review explores nurses’ oral health education, knowledge, and practices when caring for persons living with cancer. **Methods:** A systematic search of PubMed, DOSS, EMBASE, CINAHL, and Google Scholar identified 10 relevant studies. **Results:** Inconsistencies in oral care education, knowledge and practice were found among nurses caring for cancer patients. However, nurses with advanced education appear to be more knowledgeable and more likely to prioritize oral care for cancer patients. Collaboration with oral health professionals help to integrate oral health into nursing practice. **Conclusions:** Oral health practices in cancer care are critical, especially for individuals facing disparities in accessing a dental home. System, institutional, and provider-level supports are needed to enhance oral health in cancer care.

The rise in cancer diagnoses from 18.1 to 19.2 million between 2018 and 2020 represents a 6% increase worldwide. During the same period, cancer mortality rates increased by 3%, from 9.6 to 9.9 million (Ferlay et al., 2019). The increasing incidence of cancer and cancer mortality suggests a growing burden of disease and subsequent therapy on individuals, society, and healthcare systems. Oral sequelae of cancer therapy include mucositis/stomatitis, xerostomia, salivary gland hypofunction, infection, bleeding, neurotoxicity, osteonecrosis, radiation caries, dysgeusia, dysphagia, and trismus (Poulopoulos et al., 2017). Oral sequelae of cancer therapy can be debilitating to a person's ability to eat and drink, nutritional status, emotional state, self-image, and overall quality of life (Hartnett, 2015; Moore et al., 2014). A diminished oral health-related quality of life (OHRQoL) among cancer patients is well documented (Abed et al., 2019; Andreassen & Hadler-Olsen, 2023; Barkokebas et al., 2015; Niklander et al., 2017). To minimize oral sequelae, Abed et al. (2019) observed that most cancer patients adhere to oral hygiene recommendations when provided with guidance.

Despite the oral sequelae associated with cancer therapy, Paunovich et al. (2000) identified a lack of information available on the integration of oral health professionals in cancer care settings. However, a study by Epstein et al. (2007) observed that 56% of cancer care centers did not have a dental department. Therefore, it is assumed that oral health professionals are underrepresented in cancer care. Without the presence of an oral health professional on the cancer care team, nurses play an important role in oral health education and support, including routine assessment, as well as prevention and management of oral sequelae. This is especially important for persons living with cancer (cancer patients) who lack access to a dental home. Nurses are well-positioned to bridge the gap between medical and oral health care.

Basic oral health assessment, promotion, education, and intervention are within the nursing scope of practice and include the oral complications of cancer therapy (Abdo et al., 2021; Dolce, 2012). However, it is unknown how well nurses are fulfilling these entrustable professional oral health activities. This review aims to explore nurses’ oral health education, knowledge, and practice patterns when caring for cancer patients.

## Methods

An exhaustive search was conducted using the databases PubMed, CINAHL, DOSS, and Embase. Google Scholar was used as a supplementary tool to enhance and support the findings. The complete search strategy is provided in Table 1. The search strategy was refined by hand-searching the relevant studies’ reference lists until no new articles were found.

**Table 1.** Search Strategy.

Date searched	Results	Database	Search terms	Notes/comments
December 21, 2022	1,581	PubMed	(((((cancer[Title/Abstract] OR oncology[Title/Abstract] OR carcinoma[Title/Abstract] OR malignancy[Title/Abstract] OR leukemia[Title/Abstract] OR leukaemia[Title/Abstract] OR tumor[Title/Abstract] OR tumour[Title/Abstract] OR neoplasm[Title/Abstract] OR neoplastic[Title/Abstract] OR lymphoma[Title/Abstract]) AND (nurs*[Title/Abstract])) OR ((“Neoplasms”[Mesh]) AND (“Nursing”[Mesh]))) OR ((“Neoplasms”[Mesh]) AND (“Nurses”[Mesh]))) OR ((“Oncology Nursing”[Mesh]) OR “Neoplasms/nursing”[Mesh])) AND ((((“Oral Health”[Mesh]) OR “Mouth Diseases”[Mesh]) OR “Oral Manifestations”[Mesh]) OR (oral[Title/Abstract] OR mouth[Title/Abstract] OR teeth[Title/Abstract] OR saliva*[Title/Abstract] OR palate[Title/Abstract] OR mucosa[Title/Abstract] OR gingival[Title/Abstract] OR gums[Title/Abstract]))	
December 23, 2022	1358	CINAHL	S1—TI carcinoma OR AB carcinoma S2—TI leukemia OR AB leukemia S3—TI leukaemia OR AB leukaemia S4—TI tumour OR AB tumour S5—TI (neoplasms or oncology or cancer or tumor or malignancy) OR AB (neoplasms or oncology or cancer or tumor or malignancy) S6—TI neoplastic OR AB neoplastic S7—S1 OR S2 OR S3 OR S4 OR S5 OR S6 S8—TI nurs* OR AB nurs* S9— (S7 AND S8) S10— (MH “Neoplasms+”) S11— (MH “Nurses+”) S12—S10 AND S11 S13— (MH “Nursing Care+”) S14—S10 AND S13 S15— (MH “Oncologic Nursing+”) S16— (MH “Neoplasms+/NU”) S17—S9 OR S12 OR S14 OR S15 OR S16 S18— (MH “Oral Health”) OR (MH “Mouth Diseases+”) OR (MH “Oral Manifestations”) S19—TI (mouth OR oral OR teeth OR saliva* OR palate OR mucosa OR gingival OR gums) OR AB (mouth OR oral OR teeth OR saliva* OR palate OR mucosa OR gingival OR gums) S20—S18 OR S19 S21—S17 AND S20	
December 23, 2023	79	DOSS	S1—TI carcinoma OR AB carcinoma S2—TI leukemia OR AB leukemia S3—TI leukaemia OR AB leukaemia S4—TI tumour OR AB tumour S5—TI (neoplasms or oncology or cancer or tumor or malignancy) OR AB (neoplasms or oncology or cancer or tumor or malignancy) S6—TI neoplastic OR AB neoplastic S7—S1 OR S2 OR S3 OR S4 OR S5 OR S6 S8—TI nurs* OR AB nurs* S9— (S7 AND S8) S10—TI (mouth OR oral OR teeth OR saliva* OR palate OR mucosa OR gingival OR gums) OR AB (mouth OR oral OR teeth OR saliva* OR palate OR mucosa OR gingival OR gums) S11—S9 AND S10 S12—S9 AND S10 S13—S9 AND S10	
January 2, 2023	628	Embase	#1—cancer:ab,ti OR oncology:ab,ti OR carcinoma:ab,ti OR malignancy:ab,ti OR leukemia:ab,ti OR leukaemia:ab,ti OR tumor:ab,ti OR tumour:ab,ti OR neoplasm:ab,ti OR neoplastic:ab,ti OR lymphoma:ab,ti #2—nurs*:ab,ti #3—#1 AND #2 #4— “neoplasm”/exp #5— “nursing”/exp #6—#4 AND #5 #7— “oncology nursing”/exp #8— “nursing care”/exp #9—#4 AND #8 #10—#6 OR #7 OR #9 #11— “dental health”/exp #12— “mouth disease”/exp #13—oral:ab,ti OR mouth:ab,ti OR teeth:ab,ti OR saliva*:ab,ti OR palate:ab,ti OR mucosa:ab,ti OR gingival:ab,ti OR gums:ab,ti #14—#11 OR #12 OR #13 #15—#3 AND #10 AND #14	
January 12, 2023	100	Google Scholar	(cancer OR oncology OR carcinoma OR malignancy OR leukemia OR leukaemia OR tumor OR tumour OR neoplasm OR neoplastic OR lymphoma) AND nurs* AND (oral OR mouth OR teeth OR saliva* OR palate OR mucosa OR gingival OR gums)	- Millions of results—extracted the first 100

This review included primary and secondary studies investigating nurse's oral health knowledge, attitudes, and practice patterns when caring for cancer patients. All methodologies and study designs were included for studies available in full text and English language. Oral complications of chemotherapy, radiation therapy, and surgery were considered. Nurses from all demographics (age, race, ethnicity, and country) and levels of education (diploma, degree, masters) were included. This review was limited to nurses with experience caring for cancer patients but not limited to nurses working within the discipline of oncology. Non-peer-reviewed sources and studies published before the year 2000 were excluded.

The studies among the five databases were uploaded into Covidence^®^ systematic review software (Covidence—Better Systematic Review Management, n.d.) and duplicates were removed. Following the PRISMA guidelines (Page et al., 2021), three of the researchers (RD, MH, and EP) independently completed the title and abstract screening, followed by a full-text review to determine eligibility using the predefined inclusion and exclusion criteria. Conflicts were discussed until a consensus was reached. The research team pre-established the information of interest and created a data extraction tool as a framework to guide data collection.

## Results

**Figure 1.**
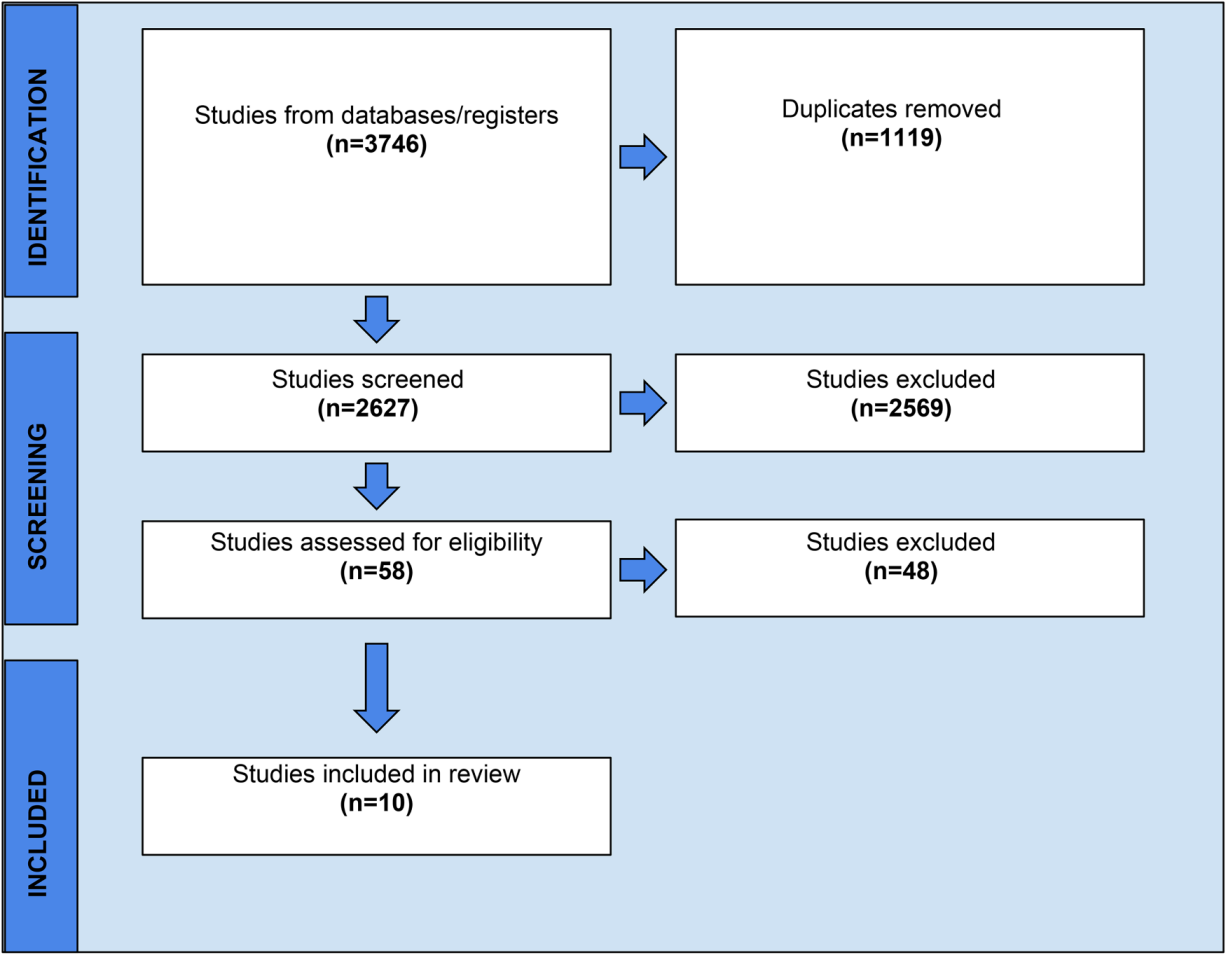
Screening and selection of articles.

Initially, 3,746 studies were identified among the five databases (Figure 1). After duplicates were removed, titles and abstracts of 2,627 studies were screened. Of the 2,627 studies, 2,569 did not meet inclusion criteria, therefore 58 studies remained for the full-text screening. Following the full-text screening, 10 studies were selected for this review (Gündogdu & Sayar, 2022; Ohrn et al., 2000; Pai et al., 2019; Pai & Ongole, 2015; Perry et al., 2015; Sharour, 2019; Southern, 2007; Suminski et al., 2017; Tewogbade et al., 2008; Wei et al., 2022).

All studies used a quantitative, cross-sectional descriptive design (Gündogdu & Sayar, 2022; Ohrn et al., 2000; Pai et al., 2019; Pai & Ongole, 2015; Perry et al., 2015; Sharour, 2019; Southern, 2007; Suminski et al., 2017; Tewogbade et al., 2008; Wei et al., 2022) (Table 2). In contrast to a self-administered questionnaire (Gündogdu & Sayar, 2022; Ohrn et al., 2000; Pai et al., 2019; Pai & Ongole, 2015; Perry et al., 2015; Southern, 2007; Suminski et al., 2017; Tewogbade et al., 2008; Wei et al., 2022), Ohrn et al. (2000) chose a semi-structured interview method to administer a questionnaire. Sharour (2019), Southern (2007), Pai and Ongole (2015), and Tewogbade et al. (2008) included knowledge testing questions. In addition to the knowledge test, Sharour (2019) observed clinical nursing practice. Various oral complications were explored in 8 of the 10 studies (Ohrn et al., 2000; Pai et al., 2019; Pai & Ongole, 2015; Perry et al., 2015; Southern, 2007; Suminski et al., 2017; Tewogbade et al., 2008; Wei et al., 2022), whereas Sharour (2019) and Gündogdu and Sayar (2022) focused on solely oral mucositis. Ohrn et al. (2000) explored nurses caring for patients with head and neck cancer, lung cancer, and hematological malignancies. Suminski et al. (2017) studied nurses caring for patients with breast cancer. Wei et al. (2022) investigated postsurgical patients with oral cancer. Perry et al. (2015) and Tewogbade et al. (2008) concentrated exclusively on pediatric oncology. The remaining studies did not specify a particular type of cancer or population (Gündogdu & Sayar, 2022; Pai et al., 2019; Pai & Ongole, 2015; Perry et al., 2015; Sharour, 2019; Southern, 2007; Tewogbade et al., 2008). The year of publication ranged from 2000 to 2022. The number of participants ranged from 20 to 235 participants. Two of the studies were conducted in Europe (Ohrn et al., 2000; Southern, 2007), three in North America (Perry et al., 2015; Suminski et al., 2017; Tewogbade et al., 2008), and five in Asia (Gündogdu & Sayar, 2022; Pai et al., 2019; Pai & Ongole, 2015; Sharour, 2019; Wei et al., 2022).

**Table 2.** Characteristics of Eligible Studies.

Authors; year	Country of origin	Study design	Study objective	Methods/ instruments	Inclusion/exclusion criteria	Sampling	Participants/setting	Stated limitations/bias
Suminski et al. (2017)	United States	Descriptive cross-sectional	To explore oncology nurses’ perceptions of their educational experiences, professional attitudes, and behavior related to providing oral healthcare education to patients with breast cancer.	- Questionnaire - Sent October 2014 and a follow-up email was sent 2 weeks later; no time frame was indicated	Practicing, fully licensed oncology nurses and nurse practitioners with experience treating patients with breast cancer	- The sampling technique was not stated - *n* = 164 (3.3% response rate)	5,000 nursing team members of the Oncology Nursing Society with experience treating patients with breast cancer were invited to participate.	- Low response rate - Potential for social desirability bias
Sharour (2019)	Jordan	Descriptive cross-sectional and observational	To evaluate oncology nurses’ knowledge and compliance with oral mucositis management guidelines.	- Phase I—knowledge test; no time frame indicated - Phase II—nurses were observed and graded based on an oral care checklist; timeframe: 2 weeks	Nurses working in an oncology unit for a minimum of 6 months with experience treating patients with oral mucositis	- Phase I: convenience sample; *n* = 140 (70% response rate) - Phase II: random sample; *n* = 20	Phase I - Nurses from oncology units, including surgical, medical, hematological, pediatric, and adult clinics were invited to participate. Phase II - A random sample of participants from phase I.	Possibility of Hawthorne effect
Ohrn et al. (2000)	Sweden	Descriptive cross-sectional	To describe the perception of education, self-rated knowledge, and attitudes towards oral care, performed oral care, and cooperation with dentistry among nurses and enrolled nurses in charge of patients with hematological malignancies, lung cancer, and head and neck cancer.	- Semi-structured interview/orally administered questionnaire - No time frame is indicated	At least 6 months of experience working with lung cancer, hematological malignancies, and head and neck cancer patients	- Sampling technique not stated - *n* = 137 (92% response rate)	All experienced nurses working in one of the 2 participating hospitals in Sweden were invited to participate.	- Generalizability is limited - Potential for social desirability
Pai et al. (2019)	India	Descriptive cross-sectional	To determine nurses’ practice and barriers regarding oral care in cancer patients undergoing chemotherapy and radiation therapy.	- Questionnaire - Timeframe: July 2013–Jan 2014	Registered nurses with a minimum of 1 year of experience caring for cancer patients undergoing therapy	- Purposive sample; *n* = 158 (79% response rate)	Nurses with oncology experience working in one of four preselected hospital sites.	- Generalizability is limited - Limitations to using a self-reporting technique
Perry et al. (2015)	United States	Descriptive cross-sectional	To examine the knowledge, perceived ability, and practice behaviors of pediatric oncology and hematology nurses in assisting with the various oral health care needs of pediatric oncology patients and to identify pediatric oncology nurses’ previous training/education, practice types, and other demographic characteristics that are related to their oral health competencies.	- Questionnaire - Timeframe: Oct 4, 2012–Oct 6, 2012	Nurses and nurse practitioners with experience working in pediatric oncology	- Convenience sample - *n* = 235 (78% response rate)	Nurses and nurse practitioners with experience and attended the Association of Pediatric, Hematology and Oncology Nurses (APHON) 36th Annual Conference and Exhibit.	- Missing data - Generalizability is limited - Did not use a validated tool
Southern (2007)	Ireland	Descriptive cross-sectional	- To describe nurses’ education and knowledge gained during their initial education in relation to oral care and oral health assessment. - To determine nurses’ self-rated knowledge of oral care and oral assessment. - To describe any differences between registered nurses and specialist oncology nurses with regard to their knowledge and levels of education in relation to oral care and oral assessment. - To identify how nurses on oncology wards manage oral care. - To explore the influences on nurses’ knowledge of oral care and performed oral care.	- Questionnaire - Timeframe: one point in time in 2003	Registered general nurses and specialist cancer nurses	- Non-random convenience sample - *n* = 72 (72% response rate)	Registered nurses and specialty cancer nurses working in one health care institution in Ireland.	- Generalizability is limited - Limitations to using a self-reporting technique
Pai and Ongole (2015)	India	Descriptive cross-sectional	To determine the nurses’ knowledge and education about oral care in cancer patients undergoing chemotherapy and radiation therapy.	- Knowledge test - Timeframe: July 2013–Jan 2014	Registered nurses with at least 1 year of experience caring for cancer patients undergoing cancer treatment	- Non-random purposive sample - *n* = 158 (response rate =79%) - 4 pre-selected hospital sites	Registered nurses working in one of the four pre-selected hospital sites Udupi and Dakshina Kannada District in Karnataka State.	- Generalizability is limited - Limitations to using a self-reporting technique
Tewogbade et al. (2008)	United States	Descriptive cross-sectional	To gain information on deficiencies in nurses’ knowledge and practice with regard to oral care of pediatric patients undergoing hematopoietic stem cell transplantation (HSCT) and cancer treatment.	- The questionnaire included knowledge testing - Timeframe: Nov 2005–Apr 2006	Nurses working in a pediatric oncology inpatient unit	- Convenience sample - *n* = 33 (response rate = 82.5%)	Nurses working in the Center for Cancer and Blood Disorders (CCBD) unit of Children's Medical Center, Dallas, Texas (CMC) who attended staff meetings.	- The sample size did not allow for statistical analysis - Single site study - Face validity of a self-reported questionnaire - The wording of some questions made responses difficult to interpret
Gündogdu and Sayar (2022)	Turkey	Descriptive cross-sectional	To explore the practices of oncology nurses in the management of chemotherapy-related oral mucositis (OM) by the Multinational Association of Supportive Care in Cancer and International Society of Oral Oncology (MASCC/ISOO) guidelines.	- Questionnaire - Timeframe: Dec 25, 2021–Jan 31, 2022	- Oncology nurses responsible for the administration of chemotherapy - Incomplete questionnaires were excluded	- The sampling technique was not stated - *n* = 157 (response rate = 47%)	Nurses working in one of the nine provinces of Turkey (Ankara, Istanbul, Izmir, Bursa, Diyarbakır, Çanakkale, Sakarya, Afyon).	- Generalizability is limited
Wei et al. (2022)	China	Descriptive cross-sectional	To assess the practicing situation of nurses in the intensive care unit (ICU) for postoperative patients with oral cancer and their need for training.	- Questionnaire - Timeframe: Sep 2020–Dec 2020	- Registered nurses and had worked in the ICU for >12 months - Nurses on leave were excluded	- Cluster sample - *n* = 173 (response rate = 10%)	- Nurses working in ICUs in one of the 11 tertiary hospitals conducting oral cancer surgery in Henan province, China.	- Generalizability is limited - Low response rate - Limitations to using a self-reporting technique

### Oral Health Education and Knowledge

Five studies reported on formal education and the correlation with nurses’ knowledge of oral health care for cancer patients (Pai et al., 2019; Pai & Ongole, 2015; Perry et al., 2015; Sharour, 2019; Southern, 2007). Sharour (2019) suggests that as the level of education increases (diploma, bachelor, and postgraduate), so does the nurses’ tested oral health knowledge and skill performance (*p* < .05). However, the overall results of the knowledge test determined that the majority of 140 nurse participants had poor knowledge of oral pathology (64.3%, *n* = 90), oral mucositis (71.4%, *n* = 100), and patient education and advice (69.3%, *n* = 97), regardless of their level of education. Sharour (2019) used a 44-item checklist when observing oral assessment, oral hygiene, patient education, and documentation. Scores of 23–44 were considered satisfactory. When observed, Sharour (2019) considered the skill performance of nurses to be unsatisfactory overall. There were significant differences (*p* < .05) in skill performance between nurses with a postgraduate degree, bachelor's degree, and a diploma degree, 28.50 ± 0.65, 26.50 ± 3.18, and 18.50 ± 4.58, respectively. Perry et al. (2015) found nurses with an oral health clinical component within their nursing program curriculum to have greater knowledge and confidence when examining the oral cavity, identifying complications and providing oral hygiene instruction (OHI; *p* < .02). Southern (2007) found differences in the self-rated knowledge scores of nurses who received “some” oral care education, compared to those who received “a lot” of oral care education during their oncology nursing training (*p* = .002). However, the majority (74.5%, *n* = 169) reported only 3 hours or less of training and no clinical requirements (60%, *n* = 140). Those with a clinical requirement had more knowledge and confidence in oral assessment and management and were more likely to assess their patient's mouth and provide OHI. The remaining two studies discuss formal education but did not report statistically significant findings (Pai et al., 2019; Pai & Ongole, 2015).

Five of the studies (Ohrn et al., 2000; Perry et al., 2015; Sharour, 2019; Southern, 2007; Wei et al., 2022) reported an association between continuing education (CE) and nurses’ knowledge of oral care for cancer patients. Specifically, Southern (2007) reported that 94.5% (*n* = 68) of nurses believe oral health CE is needed for nurses working in oncology settings. Similarly, a need for CE was reported by 86% (*n* = 118) of respondents (Ohrn et al., 2000; Perry et al., 2015). Perry et al. (2015) found CE courses improved perceived competence and confidence in all domains of practice except for oral assessment (*p* < .007). When considering how knowledge translates to practice, Ohrn et al. (2000) found nurses place a higher priority on oral care after receiving on-the-job training and mentorship from an experienced nurse or dental professional (*p* < .05). While the remaining two studies (Sharour, 2019; Wei et al., 2022) discuss CE, they did not report statistically significant findings.

### Interprofessional Collaboration, Prioritization, and Knowledge

Two of the studies (Ohrn et al., 2000; Southern, 2007) suggest greater perceived knowledge of oral care for cancer patients among nurses who work in collaboration with a dental professional. Ohrn et al. (2000) found that 79% of nurses who collaborate with dental professionals have greater perceived knowledge of pain management (*p* < .0001), oral examinations (*p* < .05), and saliva substitutes (*p* < .05), when compared to those who do not. Similarly, Southern (2007) reported that in addition to increased perceived knowledge, nurses are more likely to prioritize cancer patients’ oral health when supported by a hospital dentist (*p* = .013). The importance of collaboration with other healthcare professionals was investigated in six of the other studies (Gündogdu & Sayar, 2022; Ohrn et al., 2000; Pai et al., 2019; Perry et al., 2015; Suminski et al., 2017; Tewogbade et al., 2008); however, those studies did not report statistically significant findings.

### Age, Years of Experience, and Oral Health Practices

Among the 10 studies, three reported on nurses’ years of experience or age and their oral health practices (Ohrn et al., 2000; Perry et al., 2015; Southern, 2007). Ohrn et al. (2000) and Southern (2007) identified that younger nurses were more likely to discuss OHI (*p* < .01, *p* = .034, respectively). In addition to greater perceived oral health-related knowledge, Perry et al. (2015) reported improved practices among nurses who had additional years of experience in pediatric oncology (*p* < .05), with the exception of oral assessment practices. There were no differences in oral assessment practices based on years of experience (*p* ≥ .1).

### Knowledge and Oral Health Practices

Seven studies reported a correlation between knowledge and practice (Ohrn et al., 2000; Pai et al., 2019; Perry et al., 2015; Sharour, 2019; Southern, 2007; Tewogbade et al., 2008; Wei et al., 2022). Ohrn et al. (2000) and Southern (2007) found nurses whose perceived knowledge was greater examined the oral cavity more frequently (*p* < .05; *p* = .03, respectively). Specifically, Ohrn et al. (2000) found nurses with a greater perceived knowledge of oral status, signs, and symptoms of disease and fluoride examined the oral cavity more frequently (*p* < .05). Similarly, Southern (2007) found that nurses who self-report to have a greater knowledge of the oral signs and symptoms of abnormalities (*p* = .003) and oral care (*p* = .024) often examine the oral cavity more than once a day (*p* = .015). Furthermore, nurses who report they always create an individualized oral care plan perceive their knowledge to be greater than those who only sometimes or never created an individualized oral care plan (*p* = .042) (Southern, 2007). The remaining five studies did not report statistically significant findings (Pai et al., 2019; Perry et al., 2015; Sharour, 2019; Tewogbade et al., 2008; Wei et al., 2022).

### Oral Health Practice Patterns

When observed, Sharour (2019) considered the skill performance of nurses to be unsatisfactory despite significant differences (*p* < .05) in skill performance between nurses with a postgraduate degree, bachelor's degree, and a diploma degree. Among participants, 60% (*n* = 12) made mistakes when performing an oral assessment and the majority (90%, *n* = 80) did not use a valid and reliable assessment tool. Assessment of risk factors and prior oral health status were often overlooked (70%, *n* = 16). For interventions, the majority of nurses did not offer lip lubricant (70%, *n* = 16), high-fluoridated products (70%, *n* = 14), or saltwater rinses (65%, *n* = 13). Patient education and counseling were considered inadequate and too brief.

#### Oral Assessments

The frequency and timing of oral assessments vary greatly among nurses overall. Responses indicate that the frequency of oral assessments range from daily or during every 12 hr shift, more often than daily, on admission, before treatment or invasive procedures, before discharge, upon patient complaint/request, if treatment complications exist, symptoms were present, or never. The most common responses were daily or every 12 hr (Pai et al., 2019; Pai & Ongole, 2015), if symptoms were present (Suminski et al., 2017), and upon patient complaint/request (Pai et al., 2019; Perry et al., 2015). Studies highlight inconsistencies on the conditions that were assessed and how assessments were conducted. (Gündogdu & Sayar, 2022; Southern, 2007). Gündogdu and Sayar (2022) reported that 63% (*n* = 99) of nurses regularly used an oral assessment guide (OAG), and use of an OAG was found to be beneficial when examining the mouth (Southern, 2007)*.*

#### Oral Hygiene Instruction (OHI)

Eight of the 10 studies explored OHI for cancer patients; however, no statistically significant findings were reported (Gündogdu & Sayar, 2022; Ohrn et al., 2000; Pai et al., 2019; Perry et al., 2015; Sharour, 2019; Southern, 2007; Suminski et al., 2017; Tewogbade et al., 2008). There was a notable range in the extent that patients and caregivers were educated on oral hygiene practices, with only approximately one-third receiving OHI (Ohrn et al., 2000; Pai et al., 2019; Suminski et al., 2017; Tewogbade et al., 2008). Moreover, there were occasions when respondents offered minimal to no OHI unless patients raised concerns or encountered oral complications (Ohrn et al., 2000; Southern, 2007). When OHI was implemented, there was an emphasis on cleaning the mouth thoroughly (Ohrn et al., 2000; Southern, 2007). There were conflicting results on nurses level of confidence in providing OHI (Perry et al., 2015) and their perception of having adquate knowledge to do so (Sharour, 2019).

#### Referrals

Five studies discussed oncology nurses collaborating with or referring to dental professionals (Gündogdu & Sayar, 2022; Perry et al., 2015; Southern, 2007; Suminski et al., 2017; Tewogbade et al., 2008). Several studies indicated that more than half of nurses do not refer patients undergoing cancer therapy to a dental professional (Gündogdu & Sayar, 2022; Perry et al., 2015; Suminski et al., 2017). Alternatively, Southern (2007) found that 79.2% of patients were referred to hospital dentists.

#### Documentation

Three of the studies explored nursing documentation practices of oral sequelae (Ohrn et al., 2000; Pai et al., 2019; Southern, 2007). Southern (2007) reported the nurse participants who sometimes or always documented oral findings placed a higher degree of priority on oral care (*p* = .002). Ohrn et al. (2000) found that the majority of nurses only documented when there were changes or oral sequelae within the oral cavity. In contrast, Pai et al. (2019) found that while only 26.6% of nurses documented such findings, most nurses documented each time they provided oral care to the patient.

## Discussion

Knowledge (both actual and perceived), skill, prioritization of oral health, and practices appear to be greater with higher levels of nursing education (diploma, degree, master's level) (Sharour, 2019); however, oral health knowledge, skill, and practices seem unsatisfactory among nurses caring for cancer patients, regardless of their level of education. System, institution, and provider-level supports are needed to enhance oral health services and ensure quality care in oncology, including fostering collaboration with oral health professionals or, ideally, integrating an oral health professional into the care team. As well, oral health curriculum within nursing programs must enable graduates to be competent to perform oral health entrustable activities (Abdo et al., 2021; Dolce, 2012). Curriculum within nursing programs on oral health in oncology enhances nurses’ confidence, OHI, and the number of referrals made (Warren et al., 2022).

Formal nursing programs that dedicate more time to oral health education and include a clinical component result in nurses who graduate with more perceived knowledge. A recent systematic review reinforces this finding, highlighting a positive correlation between interprofessional education, encompassing lectures and clinical simulations, on nursing students’ oral health knowledge, attitudes, and practice patterns (Bhagat et al., 2020). Continuing oral health education appears to have a positive association with perceived knowledge and practice among nurses caring for cancer patients. Two recent reviews corroborate this finding and further underscore the positive influence that ongoing educational initiatives have on the cognition and clinical practices of nurses (Albougami, 2023; Cant & Levett-Jones, 2021). It is also noteworthy that half of the studies included in this review reported low attendance of CE courses on oral care; however, it is unclear if courses were unavailable or if they were simply not attended (Ohrn et al., 2000; Perry et al., 2015; Sharour, 2019; Southern, 2007; Wei et al., 2022). Of the studies reporting low CE attendance, a perceived knowledge deficit among nurses was also reported (Ohrn et al., 2000; Perry et al., 2015; Southern, 2007). Although there was low attendance of CE courses, most nurses felt that CE on oral health would be valuable to their practice (Ohrn et al., 2000; Pai et al., 2019; Pai & Ongole, 2015; Perry et al., 2015; Sharour, 2019; Southern, 2007; Suminski et al., 2017; Wei et al., 2022). This poses the question of whether barriers exist that may prevent nurses from furthering their education in this domain, and how the nursing profession could be further supported. For example, a lack of time was noted as a barrier to providing oral health care (Gündogdu & Sayar, 2022; Ohrn et al., 2000; Pai et al., 2019; Southern, 2007; Suminski et al., 2017). This finding is also documented throughout existing nursing literature (Albougami, 2023; Grant et al., 2021).

Although interprofessional collaboration is recognized as significant in cancer care, few nurses actually collaborate with an oral health professional (Ohrn et al., 2000; Southern, 2007). This indicates that there is room to improve multidisciplinary care coordination to comprehensively manage oral health among cancer patients. A relationship with an oral health professional is considered a facilitator to integrating oral health into nurse practitioner practice in primary care (Bhagat et al., 2020; Grant et al., 2021). Nurses oral health knowledge and capacity to send patient referrals, and patient satisfaction can be improved following participation of nurses in an interprofessional collaborative program (Kohli et al., 2021). Within formal program education, dental students and nurse practitioners report benefits of interprofessional clinical experiences within their educational programs (Janotha et al., 2019). Moreover, the integration of head, ears, eyes, nose, oral cavity, and throat (HEENOT) assessments into the examination practices of nursing students has been associated with enhanced oral health competency and a rise in referrals to an oral health professional (Haber et al., 2015). Additionally, a recent systematic review reports nurses felt more confident promoting oral health when there was interprofessional collaboration and oral health education within nursing programs (Albougami, 2023). This highlights the need for interprofessional collaboration between nursing and oral health professional programs and within primary and acute care settings.

Use of OAGs has prompted a noteworthy discourse due to the observed inconsistencies (Gündogdu & Sayar, 2022; Southern, 2007), as did the evidence of suboptimal patient education (Sharour, 2019). A study by Chen et al. (2004) recognized the benefit of assessments using an OAG during and after chemotherapy to help prevent infections and improve the quality of life in children undergoing cancer therapy. Haber et al. (2015) found that following the HEENOT examination, there was enhanced competency in oral health assessments. These findings suggest that the use of an OAG to conduct the oral cavity component of the HEENOT examination may improve oral assessment practices, and in turn, reduce the oral sequlae associated with cancer therapy.

Nurses who placed a higher value on oral care reported more consistent documentation of oral findings, compared to their counterparts who placed a lesser value on oral health (Southern, 2007). Given the increasing diversity among nurses with differing values, there is a need to explore factors contributing to one's value of oral health and how to best strengthen values among nurses. The need for consistent documentation practices among nurses is underscored by its potential to enhance communication, promote continuity of care, and ultimately contribute to favorable patient outcomes. This is especially imperative during the current healthcare crisis, resulting in nursing shortages and the need for hospitals to rely heavily on temporary staff.

A large portion of respondents never provided OHI unless prompted by patients, indicating a potential gap in proactive patient education practices (Ohrn et al., 2000; Southern, 2007). A 2015 study conducted by Levin et al. (2015) demonstrated a significant improvement in oral hygiene skills of children in a hemato-oncology department after receiving OHI sessions. Existing evidence (Levin et al., 2015; Ohrn et al., 2000; Southern, 2007) emphasizes the importance of integrating preventive OHI into the care plan for cancer patients.

### Limitations

Although a comprehensive search across multiple databases was completed, it is possible that some relevant studies were missed. As the inclusion criteria only included studies available in full-text published in English, it is possible that research published in other languages was excluded resulting in language and accessibility bias. All studies were descriptive design, therefore resulting in a lower level of evidence with less reliable findings. Most studies relied on self-reported data from nurses, which may be subject to response bias. Lastly, as the studies included in the review encompassed different types of cancer, care settings, and geographic regions, there are limitations to the transferability and generalizability of the findings.

## Conclusion

With cancer rates rising, so are oral complications of cancer therapy. Oral health practices in oncology care are critical, especially for individuals facing disparities in accessing a dental home. Yet, education, knowledge and practices are inconsistent and limited overall. Oral health curriculum within nursing programs must enable graduates to be competent to perform oral health entrustable activities. Futhermore, system and institutional-level policies are needed to support nurses in their role and enhance oral health practices and outcomes for cancer patients.

## Key Points

- Common cancer therapies frequently lead to oral sequlae that can negatively impact a patient's quality of life.
- Oral health curriculum within nursing programs must enable graduates to be competent to perform oral health entrustable activities for cancer patients.
- System and institutional-level policies and support are needed to enable nurses to fulfill their role and enhance oral health practices when caring for cancer patients.
- Enabling collaboration between oral health professionals and oncology teams or, ideally, integrating oral health professionals into the care team can improve oral health outcomes and quality of life for cancer patients.
